# Weight Watchers on prescription: An observational study of weight change among adults referred to Weight Watchers by the NHS

**DOI:** 10.1186/1471-2458-11-434

**Published:** 2011-06-06

**Authors:** Amy L Ahern, Ashley D Olson, Louise M Aston, Susan A Jebb

**Affiliations:** 1 MRC Human Nutrition Research Unit, Elsie Widdowson Laboratory, Fulbourn Road, Cambridge, CB1 9NL, UK

## Abstract

**Background:**

The scale of overweight and obesity in the UK places a considerable burden on the NHS. In some areas the NHS has formed partnerships with commercial companies to offer weight management services, but there has been little evaluation of these schemes.

This study is an independent audit of the Weight Watchers NHS Referral scheme and evaluates the weight change of obese and overweight adults referred to Weight Watchers (WW) by the NHS.

**Method:**

Data was obtained from the WW NHS Referral Scheme database for 29,326 referral courses started after 2^nd ^April 2007 and ending before 6^th ^October 2009 [90% female; median age 49 years (IQR 38 - 61 years); median BMI 35.1 kg/m^2 ^(IQR 31.8 - 39.5 kg/m^2^). Participants received vouchers (funded by the PCT following referral by a healthcare professional) to attend 12 WW meetings. Body weight was measured at WW meetings and relayed to the central database.

**Results:**

Median weight change for all referrals was -2.8 kg [IQR -5.9 - -0.7 kg] representing -3.1% initial weight. 33% of all courses resulted in loss of ≥5% initial weight. 54% of courses were completed. Median weight change for those completing a first course was -5.4 kg [IQR -7.8 - -3.1 kg] or -5.6% of initial weight. 57% lost ≥5% initial weight.

**Conclusions:**

A third of all patients who were referred to WW through the WW NHS Referral Scheme and started a 12 session course achieved ≥5% weight loss, which is usually associated with clinical benefits. This is the largest audit of NHS referral to a commercial weight loss programme in the UK and results are comparable with other options for weight loss available through primary care.

## Background

A quarter of UK adults are obese and a further 41% men and 32% women are overweight [1]. If current trends continue, almost nine in ten adults will be overweight or obese by 2050 [2]. Weight-related morbidity already places a substantial burden on the NHS, with direct costs estimated at £991-1124 million per annum [3]. Weight loss of ≥5% of initial weight is associated with reductions in health risk [4], and a number of behavioural and pharmacological treatments are available for adult weight management [5]. However, many treatment options do not offer the capacity to be delivered at the necessary scale, while in other cases the NHS may lack the resources to achieve a meaningful population-level impact [6].

NICE guidelines recommend that primary care providers should discuss with patients all treatment options meeting best practice guidelines, including commercial programmes [4]. However, at present, there is a paucity of data on the effectiveness of weight management options in primary care and providers need reliable information from which to select appropriate services [4]. Weight Watchers (WW) is a lifestyle-based group weight loss programme which offers weekly meetings at community venues, a nutritionally-balanced eating plan and advice on physical activity, aiming to produce moderate weight loss. A randomised clinical trial in the USA showed that WW led to greater weight loss over 12 months than a guided self-help programme (4.3 ± 6.1 kg vs. 1.3 ± 6.1 kg; p < .001) [7]. However, there is no published data on weight change following referral to WW by a health professional, when the treatment package is funded by the Primary Care Trust (PCT) at no cost to the patient. Such information is vital to inform evidence-based commissioning of weight management services.

## Methods

### Study Design

The aim of this observational study was a retrospective examination of weight change in NHS patients referred and funded by their primary care provider to 12 sessions of WW, at a cost of £45 per participant. Data were obtained from the WW NHS Referral Scheme database for all referral courses started after 2^nd ^April 2007 (when WW began recording objective weights for all commenced referrals) and ending before 6^th ^October 2009, and were analysed by an independent research team.

### Participants

The complete dataset contained 29,560 referral courses in which the patient commenced the referral by attending at least one WW meeting. No data was available on the total number of patients who were referred but did not attend any sessions. Referrals were excluded if there was no recorded height, gender was unknown, or if there were discrepancies between course completion, defined by WW, and number of meetings attended. Data from 29,326 referral courses (99%) were included in the final analyses.

### Measurements

The initial weight of participants was measured and recorded by WW leaders and relayed to the central database by telephone or email. Body weight was measured at each subsequent session by WW leaders and the final body weight was similarly relayed to the data base when patients either lapsed (defined as no attendance or communication from a participant for a 5 week period) or completed 12 sessions.

### Statistical Analysis

Statistical analyses were conducted using Stata/IC 10.1 software. Data were not normally distributed so medians and inter-quartile ranges (IQR) were used to represent the data. Data were analysed for all referrals attending a first meeting and for participants completing all 12 sessions of a first referral course during the audit period (completers only analysis). These analyses assumed there was no weight change for participants who attended only one session. A multiple linear regression model was used to analyse the effect of covariates on percent weight change.

### Ethics

This study was an audit. No data was collected outside of routine clinical care and all databases were anonymised. All individuals referred to WW as part of the NHS referral scheme gave consent to their data being audited.

## Results

### Baseline characteristics of referrals

Of the 29,326 referrals, 26,252 (90%) were female. Median age was 49 years (IQR 38 - 61 years). Median weight at the start of the referral course was 94.3 kg (IQR 83.7 - 107.7 kg) and median start BMI was 35.1 kg/m^2 ^(IQR 31.8 - 39.5 kg/m^2^).

### Attendance

**Figure 1:**
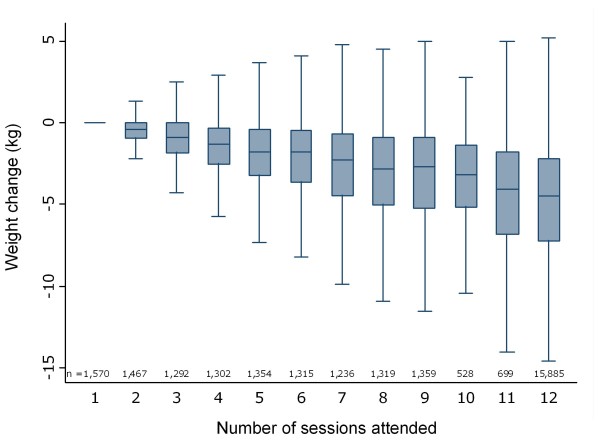
**Median and IQR of weight change (kg) for all referrals by number of sessions attended**.

Attendance for the 29,326 referral courses is shown in Figure 1. 1,570 (5%) of participants attended only 1 session and 15,885 (54%) attended all 12 sessions. Among completers, the median duration of attendance was 12 weeks (IQR 12 - 14 weeks).

### Weight change

Median weight change for all commenced referrals was -2.8 kg (IQR -5.9 - -0.7 kg), which equates to -3.1% (IQR -6.1 - -0.7%) of baseline weight. 33% of referrals resulted in ≥5% weight loss. Median weight loss increased as number of meetings attended increased, (p-trend ≤ 0.001; Figure 1).

Some courses were repeat referrals for the same patient. 11,851 (75%) of the completed 15,885 courses were the first recorded referral for an individual during the recording period. 3,088 (19%) represented a second referral and 729 (5%) a third. The remaining 217 (1%) were the fourth to eighth courses prescribed to an individual. Individuals later prescribed at least one repeat course lost more weight on their first course than those who were not prescribed a second course (t(22,517) = 37.8, p ≤ 0.001).

**Figure 2:**
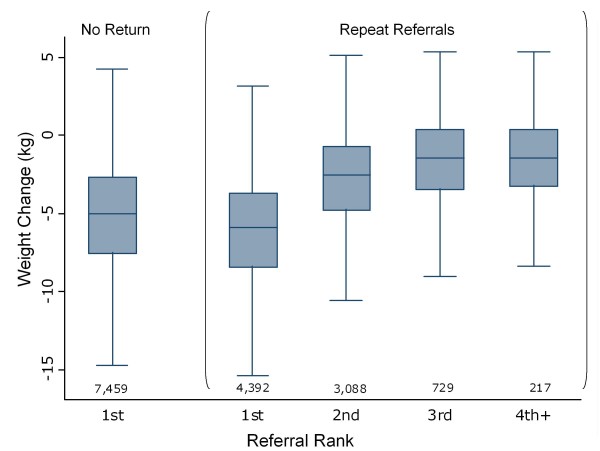
**Median weight change (kg) and IQR of completers by recorded referral rank (outliers excluded from plot)**.

Weight change by referral course number for an individual ('referral rank') is illustrated in Figure 2. Weight loss per course decreased with increasing rank, although later courses did result in small additional weight loss.

**Figure 3:**
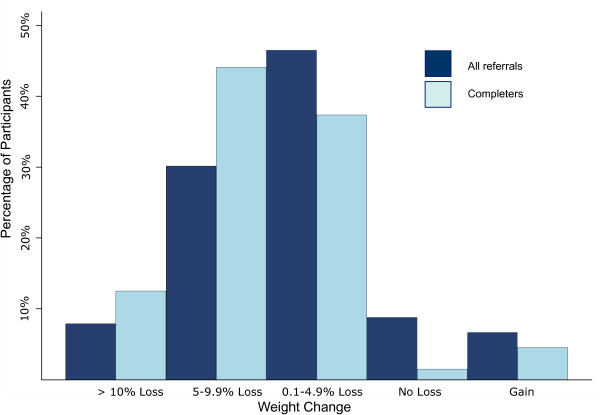
**Percentage of initial weight lost for all first recorded referrals commenced and those completing a first referral course (as a percentage of participants)**.

Median weight change for all first recorded referrals commenced (n = 22,519) was -3.6 kg (IQR -6.4 - -1.0 kg), which equates to -3.6% (IQR -6.7 - -1.1%) of baseline weight. 38% of commenced first referrals resulted in ≥5% weight loss (Figure 3).

Analysis of those who completed all 12 sessions of a first recorded referral course (n = 11,851) showed that median weight change was -5.4 kg (IQR -7.8 - -3.1 kg), or -5.6% (IQR -8.1 - -3.2%) initial weight. 57% lost ≥5% and 12% lost ≥10% of initial weight (Figure 3).

In participants completing a first referral course, median percent weight loss was greater in men than women; greater in participants over 40 y than in younger participants; and in those with a BMI between 30 and 40 kg/m^2^, relative to BMI < 30 kg/m^2^, though the absolute differences were very small (Table 1). There was statistically less weight lost as the period of time to complete the 12 sessions increased beyond the 12-week reference period, though absolute weight loss was greatest in the 5% of participants for whom final weight was recorded more than 24 weeks after baseline.

**Table 1:** Multivariate effects of covariates on median percent weight change for those completing a first referral course (median, p-value and 95% confidence interval)

		Percent Weight Change		
	N (%)	Median	P value	95% CI
Course Duration (weeks)				
12	7,972 (67)	-	-	-
13-24	3,243 (27)	0.70	< 0.001	(0.51, 0.89)
>24	636 (5)	-0.84	< 0.001	(-1.21, -0.46)
Gender				
Female	10,577 (89)	-	-	-
Male	1,274 (11)	-0.54	< 0.001	(-0.81, -0.27)
Age (years)				
< 40	2,818 (24)	-	-	-
40-49	2,606 (22)	-0.25	0.051	(0.49, 0.00)
50-59	2,612 (22)	-0.34	0.007	(-0.59, -0.09)
60+	3,815 (32)	-0.14	0.237	(-0.36, 0.09)
Start BMI				
<30	1,189 (10)	-	-	-
30-34	4,315 (36)	-0.32	0.035	(-0.62, -0.02)
35-39	3,461 (29)	-0.04	0.775	(-0.35, 0.26)
40+	2,886 (24)	0.38	0.018	(0.07, 0.70)

## Discussion

This study examined weight loss in the WW NHS referral scheme and found that a third of all commenced referrals resulted in weight loss of ≥5% initial body weight, which is expected to have clinical benefits [4]. In patients completing a first referral course, median weight loss was 5.4 kg (5.6% initial weight), with more than half losing ≥5%.

Weight loss in this programme is comparable to other studies of commercial or primary care-based weight loss programmes. For example, in the Counterweight programme 25.3% of participants lost ≥5% at 3 months, 40.2% at 6 months and 33.7% at twelve months [8]. Results are similar to a small pilot study of primary care referral to Slimming World, where participants completing a 12 week course lost 5.4 kg, with 57% losing ≥5% baseline weight. In a randomised controlled trial of commercial weight loss diets, conducted as part of a BBC series, mean weight change among self-selected participants at 2 months ranged from -3.8% to -5.5% initial weight for the various diets vs. -0.4% for controls and -4.9 to -7.3% at 6 months vs. +0.6% for controls [9].

In the present analysis over 54% of commenced referral courses were completed. High drop-out rates are usually observed in studies of obesity treatment, particularly those conducted outside of a research centre environment. In the Counterweight study, retention was 47.7% at 3 months, 30% at 6 months and 22.5% at 12 months [8] and in controlled trials, where retention is likely to be higher than in general practice, drop-out rates range from 10-80% [10].

In the UK, the NHS (primarily through PCTs) can currently purchase referral packages for 12 WW sessions, which can then be prescribed to patients at no charge. These 12 session referrals are expected to be completed over approximately 12 weeks, although many participants completed their sessions over a longer period of time. There was a trend for weight loss to diminish as the period of time to complete the 12 sessions increased beyond the 12-week reference period, perhaps reflecting declining adherence. However, absolute weight loss was greatest in the 5% of participants where the final weight was recorded more than 24 weeks after baseline, perhaps reflecting a small group of committed participants who return for a final weigh-in and who have had a longer period in which to lose weight.

Twelve weeks is a relatively short duration for a weight management programme, and data on the longer term outcomes of these referrals is necessary to establish whether this treatment has a significant impact on long term health outcomes. Research suggests that in many cases the weight lost during behavioural and pharmacological treatments is regained after treatment cessation [5,11-13]. A quarter of referrals were repeat referrals for the same patient and although weight loss diminished with repeat referrals these patients did achieve additional weight losses, suggesting that longer referrals might improve weight loss. However, increased treatment duration leads to increased costs of intervention and the longer-term impact beyond the duration of the programme still needs to be established for both commercial weight management programmes and those led by the NHS, including analyses of the cost-effectiveness of each approach.

Data was not available to calculate the cost-effectiveness in the current study. However, the current cost to PCTs of the 12 session package is £45 + VAT, which is likely to be lower than for programmes led by health professionals. One study in Australia examined the cost-effectiveness of referral to a six-month course of Weight Watchers and a weight loss treatment programme developed by a health service and concluded that neither treatment was cost effective [14]. However, this study did not base their calculations on the cost of referral packages offered to health care providers, and the authors acknowledge substantial uncertainty in their estimates and highlight the fact that, due to lack of data, they were unable to include potential health benefits additional to those resulting directly from weight loss, such as benefits derived from changes to diet and physical activity as a result of the programme. Although this study used weight loss data from a small UK study, cost data was derived from an Australian pricing structure and may not reflect the true cost-effectiveness of WW referral in the UK and other countries, and further evaluation is therefore warranted. In addition to averted healthcare costs arising from avoidance of treatment of modelled diseases, these further evaluations should also consider the considerable costs of obesity to the wider society, including time off work as a result of obesity-related illness. They should also consider comparison with alternative treatments such as individual-level primary care-based intervention.

The proportion of men referred to WW was very low, which may reflect the perception of the scheme by either prospective participants or practitioners as more suitable for women. However, weight loss in men who did attend was similar to that of women. This could suggest that the programme is equally effective in men and women, but may also reflect a tendency for men to only commence a referral if they are committed to adhering to the programme and the format is appealing to them. Further research should examine the origin of this gender bias.

Participants with a BMI between 30 and 35 kg/m lost significantly more weight compared to those with a BMI < 30 kg/m. However most of these participants will require further weight loss interventions to achieve continued reductions in excess weight. Those with a BMI >40 kg/m lost less weight than those with a BMI < 30 kg/m. Despite the large number of referrals made in this weight category, this low intensity treatment may be insufficient for this level of obesity. We speculate that the referral of heavier patients may reflect a paucity of available treatment options in primary care. Limited data from repeat referrals suggests that additional weight loss is possible, but more intensive interventions may be warranted as second-line therapy for some participants at greatest risk.

This observational study was an audit of practice-based data, so there was no control group. Further research using randomised controlled trials is needed to examine the long-term clinical outcome and cost effectiveness of WW relative to interventions led by health professionals. However, there is a dearth of research into treatment of overweight and obesity in primary care in the UK and what little there is is generally small in scale and primarily audit based or pilot data [15-17]. This study is the largest audit of a commercial weight loss programme working in partnership with the NHS and is an important first step towards building an evidence base for primary care referral to a commercial weight loss provider.

In this pragmatic analysis, the findings are limited by the data available and its quality. Weight measurements were made and recorded by WW group leaders, and were not collected for the purpose of research. Data is only available from PCTs with existing procurement contracts with WW and these are not necessarily nationally representative. In addition data is only available for those participants who 'activated' their WW packs and not those who received a referral but chose not to attend. However, it is a strength of this study that data was collected in routine care and reflects the weight loss achieved across the UK by all PCTs utilising the scheme. There are currently 67 PCTs and 1311 GPs actively referring patients to WW through their NHS Referral scheme, and 100 primary care organisations have utilised the scheme since 2005. This represents more than half the PCTs in the UK at the time.

## Conclusions

The weight loss achieved through the WW NHS Referral Scheme is comparable to other treatment options available in primary care. This intervention can be delivered on a scale that exceeds the capacity of current programmes led by NHS staff and at modest cost to the NHS. Referral to WW offers a useful first line weight-loss intervention for selected patients who are willing and able to join such groups. However, previous research has shown that in many cases the weight lost with behavioural interventions is regained after treatment cessation and future research should examine the long term outcomes of patients referred to WW through this scheme and evaluate this treatment in direct comparison to other treatment options.

## Competing interests

This data analysis presented here was funded by Weight Watchers through a grant to the Medical Research Council. None of the authors has benefitted personally from this research or received remuneration from Weight Watchers.

All authors have completed the Unified Competing Interest form at http://www.icmje.org/coi_disclosure.pdf (available on request from the corresponding author) and declare: financial support for the submitted work from Weight Watchers; SAJ receives a fee for nutrition articles, lectures and interviews from businesses linked to the Rosemary Conley organisation and the conclusions of this study may have implications for the company; no other relationships or activities that could appear to have influenced the submitted work.

## Authors' contributions

All authors contributed to the design and analysis of the study and to the drafting and revision of the final manuscript. The authors take full responsibility for the conduct of the research and the analyses and interpretation of the data. The research team had full access to the data and had the right to publish any and all data, separate and apart from the attitudes of the sponsor.

## Pre-publication history

The pre-publication history for this paper can be accessed here:

http://www.biomedcentral.com/1471-2458/11/434/prepub
